# Introduction to “Endocytosis and cellular delivery”

**DOI:** 10.1039/d6cb90009g

**Published:** 2026-03-26

**Authors:** Alexander Kros, Georgina Such, Vincent M. Rotello

**Affiliations:** a Department of Supramolecular & Biomaterials Chemistry, Leiden Institute of Chemistry, Leiden University Leiden 2333CC The Netherlands A.kros@chem.leidenuniv.nl; b School of Chemistry, University of Melbourne Parkville VIC 3010 Australia gsuch@unimelb.edu.au; c Department of Chemistry, University of Massachusetts at Amherst Amherst MA 01003 USA rotello@chem.umass.edu

## Abstract

Alexander Kros, Georgina Such, and Vincent Rotello introduce the *RSC Pharmaceutics* and *RSC Chemical Biology* themed collection on Endocytosis and cellular delivery.
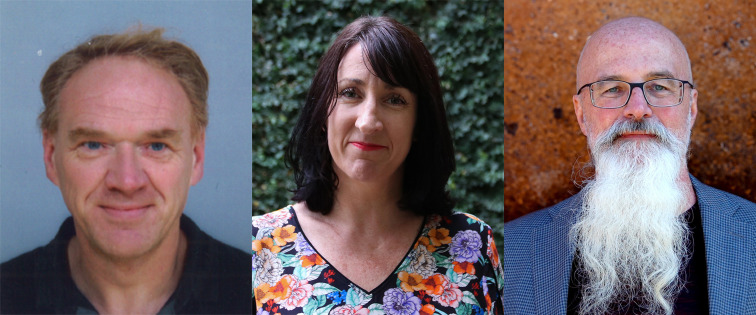

The therapeutic potential of biologics such as nucleic acids or proteins remains largely untapped due to the need for these therapeutics to transverse multiple biological barriers to be effective. Delivery systems for biologics often use nanotechnology, but many aspects of this technology remain poorly understood. To access the potential of biologics delivery, it is important that we design synthetic and biological nanosystems that can precisely control their interactions with targeted cells and facilitate efficient cellular uptake. This control is critical for maximizing payload and minimizing the off-target side-effects of the cargo, and remains an ongoing challenge. Intracellular localization is another major hurdle, with a particular impediment being the ability to access the cytosol, and hence other targets inside the cell such as the nucleus. It is essential that researchers integrate innovative materials design with rigorous studies of how structural features at the molecular level influence interactions with biological barriers. This combined approach will accelerate the development of design rules that enable more effective navigation of nanomaterials through complex biological environments.

In this collection we bring together a range of studies highlighting how the properties of nanomaterials can play an important role in governing biological interactions and thus the effectiveness of therapeutic delivery. Nagaraj *et al.* provide a review covering advances in direct delivery to the cytosol, including a wide range of strategies for protein and nucleic acid delivery.^1^ Hughes *et al.* present a clustering strategy using trastuzumab-decorated polymer nanoparticles. These studies show that rapid clustering of HER2 receptors by these nanoparticles induces rapid internalisation through endocytosis.^2^ This work uses elegant optical and electron microscopy to closely explore the clustering and uptake processes.

The shape of nanoparticles provides an important determinant for cellular uptake. Gupta *et al.* explore the role of nanoparticle shape for hydroxyapatite uptake in osteosarcoma cells.^3^ Using spherical, rod-like, and needle-shaped nanoparticles, the investigators found that rod-shaped nanoparticles were most efficiently endocytosed. This enhanced endocytosis of rod-like particles was manifested by increased cytotoxicity.

A critical component of the therapeutic challenge is avoiding the body's natural defense processes to achieve delivery to cells of interest. Younis *et al.* discuss how the protein corona formed around lipid nanoparticles can be used to direct these carriers to organs and tissues.^4^ Using the body's natural processes as a tool to enhance delivery efficiency is an important strategy for enhancing function which relies on scientists being able to understand and control these processes. Finally, Barendrecht *et al.* use an “unclicking” strategy to activate nanobody-targeted cytokines, obviating loss of activity issues arising from targeting.^5^

This themed collection provides a glimpse at the tools researchers are using to improve the delivery of biologics and nanoparticles. We believe these studies highlight a range of approaches that are critical to enhancing delivery efficiency, including materials design, harnessing the body's own defenses, and the development of new advanced biophysical tools. We hope that these articles stir your interest in understanding how delivery efficiency can be improved and look forward to the new tools and approaches you will bring to the field.
